# Wnt and β-Catenin Signaling in the Bone Metastasis of Prostate Cancer

**DOI:** 10.3390/life11101099

**Published:** 2021-10-16

**Authors:** Zachary Kaplan, Steven P. Zielske, Kristina G. Ibrahim, Frank C. Cackowski

**Affiliations:** 1 College of Literature, Science, and the Arts, University of Michigan, Ann Arbor, MI 48109, USA; zkaplan@umich.edu; 2 Department of Oncology and Karmanos Cancer Institute, Wayne State University School of Medicine, Detroit, MI 48201, USA; steven.zielske@wayne.edu (S.P.Z.); KristinaIbrahim@wayne.edu (K.G.I.)

**Keywords:** Wnt, β-catenin, bone metastasis, prostate cancer, Wnt5A, breast cancer, DKK1

## Abstract

Wnt family proteins and β-catenin are critical for the regulation of many developmental and oncogenic processes. Wnts are secreted protein ligands which signal using a canonical pathway, and involve the transcriptional co-activator β-catenin or non-canonical pathways that are independent of β-catenin. Bone metastasis is unfortunately a common occurrence in prostate cancer and can be conceptualized as a series of related steps or processes, most of which are regulated by Wnt ligands and/or β-catenin. At the primary tumor site, cancer cells often take on mesenchymal properties, termed epithelial mesenchymal transition (EMT), which are regulated in part by the Wnt receptor FZD4. Then, Wnt signaling, especially Wnt5A, is of importance as the cells circulate in the blood stream. Upon arriving in the bones, cancer cells migrate and take on stem-like or tumorigenic properties, as aided through Wnt or β-catenin signaling involving CHD11, CD24, and Wnt5A. Additionally, cancer cells can become dormant and evade therapy, in part due to regulation by Wnt5A. In the bones, E-selectin can aid in the reversal of EMT, a process termed mesenchymal epithelial transition (MET), as a part of metastatic tumorigenesis. Once bone tumors are established, Wnt/β-catenin signaling is involved in the suppression of osteoblast function largely through DKK1.

## 1. Introduction

**Figure 1:**
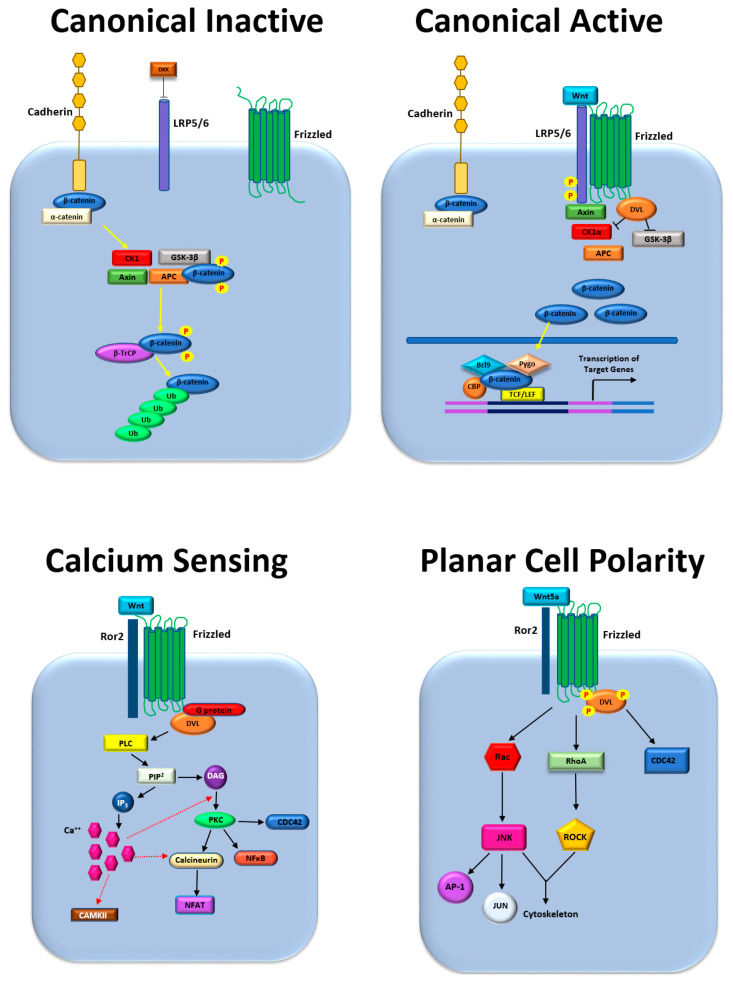
Wnt and β-catenin signaling pathways: Upper left, canonical pathway in the inactive state; Upper right, canonical pathway in the active state. Lower left, non-canonical calcium-sensing pathway; Lower right, non-canonical planar cell polarity pathway.

Signaling pathways involving proteins of the Wnt family of secreted protein ligands and the adherens junction component and transcription cofactor, β-catenin (a product of the *CTNNB1* gene), play significant roles in cancer metastasis and other oncogenic processes. These pathways involve the complex interactions between 19 ligands of the Wnt family binding to 10 receptors of the Frizzled family, with co-receptors including RYK, LRP5, and ROR2. Wnt ligands share 27% to 83% of the same amino acid sequence and contain 23 or 24 conserved cysteine residues. All of the Wnt ligands are similar in size and are held in their folded states by multiple disulfide bonds. Included among the Wnt ligands is Wnt3a, typically a ligand of the canonical pathways, and Wnt5a, typically a ligand of the non-canonical pathways [1]. Signaling downstream of the Frizzled receptors occurs via two major modes or pathways: canonical, which involves β-catenin, and non-canonical, which signals independently of β-catenin (Figure 1). In the canonical signaling pathway, the binding of a Wnt ligand to a Frizzled receptor releases β-catenin from a cytoplasmic destruction complex, which allows it to translocate to the nucleus and act as a transcription cofactor. Conversely, in non-canonical Wnt signaling, binding of a Wnt ligand to a Frizzled receptor initiates signaling most commonly through either (1) a calcium-sensing pathway downstream of phospholipase C, or (2) the planar cell polarity pathway (PCP) involving the cytoskeleton and associated small GTPases, including RHOA and RAC1. The non-canonical pathway may also proceed through signaling involving protein kinase C (PKC), calcium/calmodulin-dependent protein kinase II (CaMKII), or the nuclear factor of activated T cells (NFAT). A detailed discussion of the biochemistry and molecular biology of Wnt signaling is beyond the scope of the current work but has been the subject of multiple excellent reviews [2,3]. Here, we describe how Wnt and/or β-catenin signaling regulates prostate cancer metastasis to the bone and its growth in this new anatomic site. Although we focus our discussion on prostate cancer, we use examples from breast cancer and some other malignancies to illustrate key concepts.

**Figure 2:**
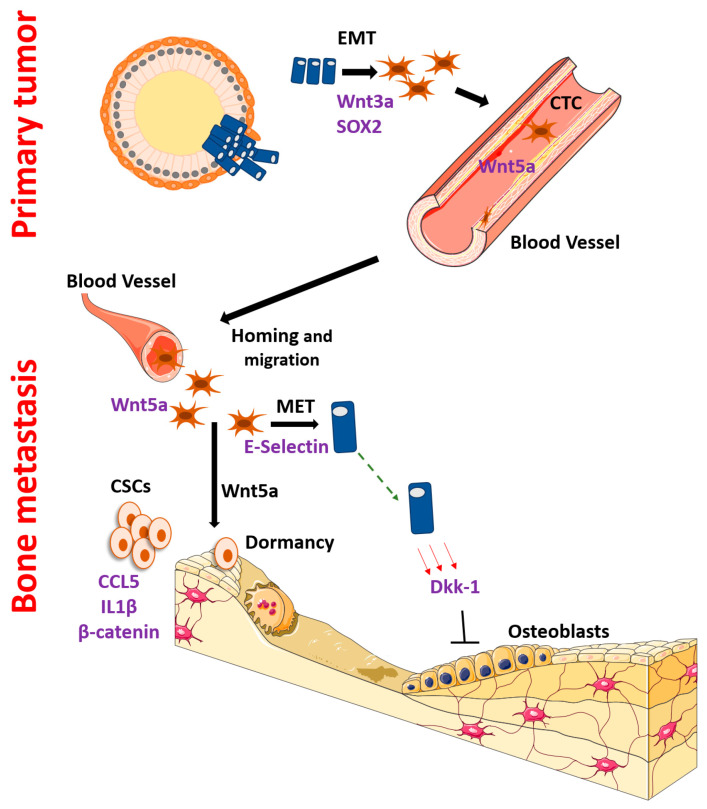
Key steps and molecules involved in the regulation of the bone metastatic cascade by Wnt and/or β-catenin signaling. Top, events at the primary tumor site; Bottom, events at the bone metastatic site.

Cancer metastasis to the bone involves a series of related processes, most of which are regulated by Wnt and/or β-catenin signaling to some extent. Critical to successful metastatic spread is that cells maintain a degree of stemness and the ability to undergo tumorigenesis at the sites of dissemination. Cells often lose epithelial properties and gain mesenchymal characteristics (epithelial to mesenchymal transition/EMT) as they detach from the primary tumor to migrate and disseminate and then revert to a more epithelial phenotype (mesenchymal to epithelial transition/MET) when they take residence in the metastatic site. Taking up residence in sites such as the bone requires complex interactions with bone, mesenchymal, and hematopoietic cells to remodel the new microenvironment, which aids tumor growth but causes dysfunction of the bone. In addition, metastasis requires the specific migration of cells into circulation and into tissues where they can remain dormant for years before the formation of gross metastatic tumors. Each step of this process, and the cell transitions undergone, have been shown to be influenced by the β-catenin pathway. Please refer to Figure 2 for a diagram of the highlights of this regulation. In this review, we concentrate on studies that have studied bone metastases directly but also include studies that do not address bone metastases directly, in order to allow an illustration of the entire bone metastatic cascade. An understanding of these processes could yield many therapeutic opportunities, especially because Wnt/β-catenin was previously thought of as not druggable; however, therapeutic development targeting the pathway has met with much more success recently [4]. Therefore, we expect the regulation of prostate cancer bone metastases by Wnt and β-catenin to be a popular and fruitful area of research in the future.

## 2. Bone Metastasis Regulated by Wnt and β-Catenin

Below we describe cell biologic processes that carcinoma cells undergo in order to disseminate and grow in the bone, including migration, phenotypic plasticity, dormancy and tumorigenesis. Although we separated these topics for ease of presentation, there are many overlaps between them. For example, cancer stem-like cells have overlapping characteristics with mesenchymal phenotype (EMT) cells, and dormant disseminated tumor cells often upregulate the expression of transcription factors important for stem cells. Thus, we encourage the reader to consider how Wnt and/or β-catenin signaling might regulate more than one of these subprocesses to allow bone metastasis formation and growth.

## 3. EMT, Initial Dissemination and Homing

In order to seed bone metastases, a cancer cell must first loosen attachments with the surrounding cells, migrate, and enter the blood stream. At the same time, epithelial cancer cells often assume characteristics of mesenchymal cells (EMT), in part regulated by Wnt and/or β-catenin signaling. Nearly half of patients with prostate cancer have a translocation between the TMPRSS2 locus and ERG or another ETS family transcription factor, which places the transcription factor under the control of androgens [5,6]. In this large subset of prostate cancers, EMT is also regulated by β-catenin signaling. TMPRSS2 translocation-positive patients have an increased expression of Wnt receptors, especially FZD4. The upregulation of this Wnt receptor leads to oncogenic effects including EMT and decreased cell adhesion [7]. Curiously, in their work, Gupta and colleagues showed the upregulation of Wnt target genes and the activity of canonical β-catenin transcription factors TCF and LEF in a reporter assay but did not observe the upregulation of β-catenin activity itself. Further work also showed direct binding of the TMPRSS2-ERG protein to the promotor of the EMT transcription factor, ZEB1, thus illustrating the complexities of Wnt and β-catenin pathways [8]. Furthermore, Wnt and β-catenin regulate EMT in prostate cancers without TMPRSS2-ERG family translocations. For example, the transcription factor SOX2 binds to the promoter region of β-catenin to mediate EMT in the DU145 prostate cancer cell line, which lacks a TMPRSS2-ERG translocation [9], and also in breast cancer cell lines [10]. The expression of SOX2 negatively correlated with the expression of E-cadherin and positively correlated with α-SMA, a mesenchymal marker protein. Although TGF-β is often an important regulator of EMT, it was not involved in these model systems [10].

Similarly, in using a mouse model of HER2^+^ breast cancer, Harper and colleagues illustrated the importance of canonical β-catenin for the initial spread of cancer cells they termed “early disseminated cancer cells” (eDCCs) [11]. These cells showed more membrane/adherens junction-associated (inactive) β-catenin and had higher amounts of β-catenin-mediated transcription than the bulk cancer cell population. They had a low level of the epithelial marker E-cadherin (CDH1) and a high level of the EMT marker Twist1, but maintained a partial epithelial character including an expression of epithelial cytokeratins. These eDCCs were initially dormant and had low levels of phosphorylated p38, but were later able to escape dormancy and form metastases. Breast cancer cells that did not respond to the HER2-targeted drug, trastuzumab, showed an upregulation of Wnt3A when compared to normal breast cancer cells. The resistant cells also had higher levels of N-cadherin, which promotes EMT and deters MET [12,13]. This correlation provides evidence that Wnt signaling potentially promotes EMT-like phenotypes in trastuzumab-resistant breast cancer cells. In keeping with its typical role as a canonical ligand, Wnt3A acted through the nuclear translocation of β-catenin [12]. The knockdown of Wnt3A by siRNA increased expression levels of E-cadherin and decreased the expression of Slug and Twist, which promoted the reversal of EMT, i.e., mesenchymal epithelial transition (MET). Additionally, β-catenin can also transmit signals from other upstream molecules other than Wnt ligands and Frizzled receptors. Wu et al. showed that PI3K can signal through GSK3β to β-catenin to induce ZEB1 expression, EMT and bone metastases in urothelial (bladder) carcinoma [14].

Because bones do not have lymphatics, almost all (if not all) cancer cells must metastasize to the bone through blood vessels. While in blood, cancer cells are often referred to as circulating tumor cells (CTCs). Especially for prostate cancer, investigators have shown the importance of the non-canonical ligand Wnt5A for CTC function. In a single cell RNA sequencing study of prostate cancer patient CTCs, Miyamoto and colleagues identified Wnt5A as a key regulator, especially for antiandrogen resistance [15]. Similarly, using a multiplex qRT-PCR approach, Singhal et al. found that Wnt5A was one of the three genes expressed in CTCs that independently predicted overall survival in metastatic castration-resistant patients [16]. However, while these studies showed the importance of non-canonical Wnt signaling in CTCs, they did not directly prove that there is a role for Wnt signaling in bone metastases.

In an extension of these studies, Wang et al. showed that Wnt5A was partially responsible for the anatomic distribution of bone metastases in mouse models [17]. To further dissect this mechanism, they showed that JNK, FZD4, and FZD8 were partially responsible for prostate cancer cell migration in this model. Previously, others found that both canonical and non-canonical Wnt signaling was increased in metastatic vs. localized prostate cancer [18]. They also found that the transcription factor PITX2 was especially upregulated in bone metastases as compared to soft tissue metastases, and that PITX2 was important for prostate cancer migration stimulated by the non-cononical ligand, Wnt5A [18]. Recently, Tseng and co-workers found that the non-canonical Wnt receptor ROR2 suppressed prostate cancer bone metastasis through PI3K signaling in model systems and was inversely correlated with bone metastasis in patient samples [19]. In addition, canonical signaling is important for the homing of prostate cancer to the bone as well. Li and coworkers showed that FZD8 promotes prostate cancer migration through β-catenin and that FZD8 knockdown inhibited bone metastases, specifically [20]. Specifically, they reported that the unusual expression of FZD8 in prostate cancer resulted in the hyperactivation of Wnt signaling by triggering a positive feedback loop of Wnt3A, a ligand of the canonical pathway that causes bone metastasis.

## 4. Dormancy, Recurrence and Mesenchymal Epithelial Transition

Cancers, especially prostate and breast cancers, can disseminate early in the disease process and lie dormant for years or decades, then relapse at distant sites such as the bone. Part of this process of recurrence can be a reversal of some of the mesenchymal properties that the cancer cells acquired at the time of initial dissemination, a process termed mesenchymal epithelial transition (MET). As prostate or other cancer cells arrive in the bone marrow, they often share similar niches as hematopoietic stem cells, which have been used as a model for understanding cancer dormancy and recurrence [21]. Here, they are exposed to a milieu of pro- and anti-proliferation factors. The maintenance of HSCs in a quiescent state in the bone marrow has been shown to be regulated by Wnt5a [22]. Similarly, a recent report found that Wnt5A induces the dormancy of prostate cancer cells in bone [23]. Although Wnt5A is often a non-canonical ligand, here it acted by the inhibition of β-catenin (i.e., canonical signaling) through a novel mechanism. Osteoblasts in the endosteal niche secreted Wnt5A to bind to ROR2 and activated the ubiquitin ligase SIAH2, which led to the degradation of β-catenin. These dormant cells were also resistant to the commonly used prostate cancer chemotherapy drug, docetaxel. Therefore, this work illustrates how, paradoxically, dormancy can work to the advantage of a cancer by protecting the disseminated cells from treatment and other stressors. Wnt signaling is also important for dormancy induction in breast cancer. As discussed above in the EMT section, canonical β-catenin signaling was critical for early dissemination and initial dormant behavior. These early disseminated cells had a partial EMT phenotype and were dormant upon arrival in the bone marrow. This dormant behavior was characterized by the activity of p38 MAPK, a key regulator of dormancy in multiple cancers [11]. Similarly, the reversal of EMT, mesenchymal epithelial transition (MET), can also be partial. Using both breast and prostate cancer models, Esposito and colleagues found that E-selectin binds to disseminated cancer cells in the peri-vascular niche of the bone microenvironment and induces MET to promote bone metastasis [24]. However, this was not the usual type of MET, in that the usual transcription factors such as ZEB1 and SNAI1 were not affected. Rather, it was a partial MET process that induced canonical β-catenin signaling to induce E-selectin glycosylation and SOX2 and SOX9 expression to stimulate stem-like properties. This was through the expression of Wnt repressors such as DKK1, CTGF, and CYR61, which are all mesenchymal-related genes.

## 5. Stemness and Tumorigenesis

As discussed immediately above, dormancy and stemness are related and overlapping processes. Likewise, Wnt/β-catenin signaling is critical for stemness and metastatic tumorigenesis in many malignant contexts, including bone metastases. Many of the studies discussed below did not study bone metastases specifically, but may provide invaluable insight into prostate cancer metastases in the bone.

CSCs can be induced through Wnt and/or β-catenin signaling pathways through the interaction with immune cells, which could be viewed as a stressor in the new environment. Wnt5A was found to recruit and regulate bone marrow macrophages through the subsequent secretion of CCL2 and BMP6, which aided in the development of prostate cancer castration resistance [25]. Hwang et al. subsequently showed that CCL5 secreted by macrophages stimulated the formation of prostate cancer CSCs through a β-catenin-dependent mechanism [26]. CSCs were assayed by dual positivity for CD133 and CD44 or Aldefluor positivity, in addition to functional assays in vitro and in vivo. They proposed that CCL2 acted through binding to CCR5, which subsequently stimulated β-catenin activity, which then stimulated STAT3 transcription. Similarly, in breast cancer, the inflammatory mediator IL-1β secreted by bone marrow also stimulates CSC formation and bone metastatic tumorigenesis in a β-catenin-dependent fashion [27]. In this system, IL-1β activates NFKB/CREB signaling and Wnt ligand production, which stimulates colony formation.

Wnts and β-catenin also stimulate CSCs in bone metastases through non-immune-dependent mechanisms. CD24 induces metastasis to bone and cancer stemness by activating Wnt/β-catenin signaling in prostate cancer in vivo. FH535, a Wnt signaling inhibitor, reduces prostate cancer cell migration in vitro which corroborates the notion that CD24-mediated Wnt/β-catenin signaling controls cell migration and stemness. There is a positive correlation between the CD24 expression of Wnt-mediated bone metastasis of prostate cancer cells in vitro and in vivo [28]. Recently, Pan and colleagues reported a novel mechanism for prostate cancer CSC regulation [29]. Endothelial cell-specific molecule 1 (ESM1), normally a secreted proteoglycan, stimulated the formation of prostate cancer CSCs through β-catenin when localized in the nucleus. In turn, β-catenin stimulated the nuclear localization of ESM1. Stably overexpressing ESM1 resulted in the increased spheroid formation and enhanced self-renewal ability of PC3 cells in vitro. Similarly, when ESM1 was silenced in PC3 cells, spheroid formation decreased but was restored back to normal levels when ESM1 was rescued. When the group tested tumorigenicity in vivo, it was discovered that tumors overexpressing ESM1 grew larger than tumors injected with an empty vector control in immunodeficient mice. Furthermore, the presence of stem cell markers CD44 and CD133 was increased in ESM1-overexpressing cells versus the controls [29]. In another example of prostate cancer CSC formation stimulated by canonical signaling, Li et al. found that low levels of the circadian rhythm gene PER3 stimulated CSC formation through β-catenin using ALDH and CD44 positivity to define CSCs, in addition to functional assays [30]. Curiously, PER3 signaled to β-catenin through another clock-related gene, BMAL1. Overall, there are multiple mechanisms through both canonical and non-canonical Wnt pathways that regulate the formation of CSCs in prostate cancer.

## 6. Interactions with the Bone Microenvironment

After cancer cells have arrived in the bone and have formed macroscopic tumors, they interact with host bone cells to aid cancer growth, but unfortunately impair normal bone function. Much of this impairment is through the inhibition of the formation and/or the function of osteoblasts. DKK1, an inhibitor of Wnt/β-catenin signaling, stimulates osteoclasts and inhibits the formation and differentiation of osteoblasts [31]. DKK1 expression is directly linked to the production of osteolytic lesions in animal models of metastatic breast and prostate cancers. Wnt/β-catenin signaling continues in osteolytic cell lines such as PC3 and MDA-MB-231 because they express the DKK1 receptors, Kremen1 and Kremen2, at much lower levels than osteoblastic cell lines [32]. Noggin, similar to DKK1, acts as an antagonist to osteoinductive Wnt proteins in osteolytic cancer cells, and as expected, noggin is not expressed in osteoinductive cancer cell lines. Therefore, the expression of noggin and DKK1 has deleterious effects on the osteoblast response in the bone microenvironment [33,34]. In breast cancer cells, another antagonist of Wnt/β-catenin, sclerostin, inhibits osteoblast differentiation. The cancer cells secrete sclerostin which interacts with osteoblasts in the bone microenvironment. Sclerostin is typically secreted by osteocytes, but breast cancer cells gain the ability to secrete the inhibitor when in the bone marrow niche by activating RUNX2/CBFβ signaling [35].

Investigators have also worked out mechanisms involving multiple mediators working in concert to aid the growth of bone metastasis, but impair normal bone function. Dai et al. found that prostate cancer cells induce osteoblast differentiation in vitro through both canonical and non-canonical Wnt signaling and through BMP-dependent and BMP-independent mechanisms [36]. Wnt3A and Wnt5A stimulated BMP4 and BMP6 expression, which was blocked by DKK1. Noggin and DKK1 synergistically inhibited osteoblast differentiation induced by the prostate cancer cell-conditioned media. As expected, Wnt3A signaling was dependent on β-catenin, whereas Wnt5A signaling was dependent on JNK1. Non-coding RNAs are also involved in the effects of Wnt signaling on osteoblasts. For instance, miR-218 activates osteoblast differentiation and promotes osteogenesis by stimulating a positive Wnt signaling loop. Additionally, miR-218 downregulates three different Wnt inhibitors: sclerostin, DKK2, and secreted Frizzled-related protein2, and is also upregulated by Wnt signaling which forms a positive feedback loop. In metastatic breast cancer cells, miR-218 promotes osteomimicry by enhancing Wnt signaling and the expression of osteoblastic genes relating to homing and growth [37].

Furthermore, cancers can induce changes in the bone before any cells actually arrive at the metastatic site. Using the TRAMP-C1 model of localized prostate cancer, Ardura and colleagues found that tumors in the prostate increased osteoclast formation and function. Osteoblast formation was also increased through a β-catenin-dependent mechanism [38]. They found that these effects were predominantly through the secretion of Spondin-2. They found increased cancer cell adhesion in vitro and ex vivo, which might translate to the creation of a pre-metastatic niche more hospitable for bone metastasis formation. Additionally, prostate cancer cells in the bone microenvironment induce osteoblast differentiation through the PKC noncanonical pathway activated by Wnt7B. The knockdown of Wnt7B in C4-2B cells resulted in a decrease of the mRNA expression of the osteoblast differentiation markers, ALP and BSP, in the ST2 cells they were co-cultured with. When Wnt7B was overexpressed in C4-2B cells, co-cultured ST2 cells had increased ALP activity, mineralization, and ALP and BSP mRNA levels. The results of these experiments were then tested and confirmed with two other cell lines, LNCaP and LAPC4 [39].

Lastly, of particular interests to patients, Wnt signaling is also important for the transmission of bone pain [40]. He and colleagues found that Wnt5b and the co-receptor RYK had increased expression in the (sensory) dorsal root ganglia of bone tumor-bearing mice. Wnt5b increased pain sensation and RYK knockdown decreased indicators of pain. Furthermore, the process was blocked by a CaMKII inhibitor, thus indicating a non-canonical calcium-sensing pathway of Wnt signaling.

## 7. Conclusions

Wnt and β-catenin signaling pathways regulate multiple processes that impact bone metastasis. Microenvironmental cues act through these pathways to drive cells toward metastatic sites, maintain stemness and dormancy, and eventually drive cells to reactivate and form metastatic tumors. If any step of this process can be thwarted therapeutically, there is the potential to disrupt the seeding of metastatic tissues, to maintain dormancy indefinitely, or to eliminate the stemness/tumorigenic property of cells. Therefore, with the likely upcoming introduction of clinically effective drugs to target these pathways, Wnt and β-catenin pathways hold immense promise for the treatment of metastases to the bone and other sites. Furthermore, we expect that much biology is yet to be uncovered. For example, much of the existing work is centered on only three of the nineteen Wnt ligands: Wnt3A, Wnt5A, and Wnt7B. Future studies could uncover roles of additional ligands, which would improve our understanding of the regulation of bone metastases by Wnt and β-catenin signaling, and offer additional targets for pre-clinical and clinical approaches.

## Data Availability

Not applicable.
